# Quality of life assessment as a predictor of survival in non-small cell lung cancer

**DOI:** 10.1186/1471-2407-11-353

**Published:** 2011-08-15

**Authors:** Donald P Braun, Digant Gupta, Edgar D Staren

**Affiliations:** 1 Cancer Treatment Centers of America® at Midwestern Regional Medical Center, Zion, IL, 60099, USA

## Abstract

**Background:**

There are conflicting and inconsistent results in the literature on the prognostic role of quality of life (QoL) in cancer. We investigated whether QoL at admission could predict survival in lung cancer patients.

**Methods:**

The study population consisted of 1194 non-small cell lung cancer patients treated at our institution between Jan 2001 and Dec 2008. QoL was evaluated using EORTC-QLQ-C30 prior to initiation of treatment. Patient survival was defined as the time interval between the date of first patient visit and the date of death from any cause/date of last contact. Univariate and multivariate Cox regression evaluated the prognostic significance of QoL.

**Results:**

Mean age at presentation was 58.3 years. There were 605 newly diagnosed and 589 previously treated patients; 601 males and 593 females. Stage of disease at diagnosis was I, 100; II, 63; III, 348; IV, 656; and 27 indeterminate. Upon multivariate analyses, global QoL as well as physical function predicted patient survival in the entire study population. Every 10-point increase in physical function was associated with a 10% increase in survival (95% CI = 6% to 14%, p < 0.001). Similarly, every 10-point increase in global QoL was associated with a 9% increase in survival (95% CI = 6% to 11%, p < 0.001). Furthermore, physical function, nausea/vomiting, insomnia, and diarrhea (p < 0.05 for all) in newly diagnosed patients, but only physical function (p < 0.001) in previously treated patients were predictive of survival.

**Conclusions:**

Baseline global QoL and physical function provide useful prognostic information in non-small cell lung cancer patients.

## Background

Lung cancer is the most common cancer in the United States in terms of incidence and mortality with 219,440 new cases and 159,390 deaths in 2009 [1]. Patients with lung cancer experience a variety of distressing symptoms, many of which begin prior to diagnosis and continue throughout the course of the disease and its treatment, adversely affecting functional status and quality of life (QoL) [2,3]. The vast majority of patients, especially those with advanced disease, do not have curative treatment options and therefore, the goal of therapy for such patients is prolongation of survival without negatively impacting QoL [4]. Unfortunately, differences in survival time across the spectrum of available treatments for advanced lung cancer are modest. Thus, treatment for lung cancer which offers the potential for prolonging patient survival must always be judged in the context of its effects on patient QoL [5-8].

Since every patient's QoL is multidimensional, consisting of physical, functional, psychological, social, and spiritual domains [5], there is a growing consensus that treatment efficacy should be judged by its effects on both quantity and quality of life. This has led to the inclusion of QoL assessment as a primary endpoint in all types of clinical trials along with the traditional endpoints of tumor response and survival. There is general agreement in the medical and scientific research communities that patients are the best source of QoL information, especially when these patient-reported outcomes can be defined scientifically and measured with validated tools.

Extensive data in the literature have shown that QoL tools measuring the activities of daily life can predict survival in several different types of cancers independent of the extent of the disease and other clinical prognostic factors [9-27]. These studies have used different QoL instruments which can be broadly classified into generic and targeted [28]. Generic QoL instruments tend to ask questions general enough for broad applicability, including for use among individuals in good health. On the other hand, targeted measures may focus on specific symptoms that are common to a particular cancer or to its treatment. There is no "gold standard" cancer QoL questionnaire available and the choice of selecting a QoL questionnaire for a particular study is governed for the most part by the research goals of that study.

The positive relationship between QoL and survival as described above has not been universally observed in the literature, with some studies founding no association between QoL and survival [29-31]. As a result, there is no unanimity on the prognostic role of QoL in cancer. These conflicting and inconsistent results in the literature could perhaps reflect the different methodologies used to analyze the data (as an example by selecting different cut off values for variables), different patient populations investigated, different QoL questionnaires used, or alternatively, different selection of covariates to be included in the regression analysis [32]. Moreover, it is currently unclear why QoL may be prognostic, and it has been hypothesized that, in patients with micrometastatic disease, there is production of tumor factors that affect general health and that patients perceive this and report it in terms of poor QoL before it is clinically or radiologically apparent [33]. Therefore, preclinical progression of disease after curative treatment may be associated with worsening of QoL, which may be assessed with self-reported QoL measures [33]. It is possible that QoL presumably captures those aspects of disease severity that may not be apparent in the observer-rated performance status or tumor burden [34]. In the light of the above, we investigated whether QoL can predict survival in non-small cell lung cancer patients treated at a community hospital comprehensive cancer center.

## Methods

### Study Sample

We examined 1194 histologically confirmed non-small cell lung cancer patients treated at Cancer Treatment Centers of America^® ^(CTCA) at Midwestern (MRMC) and Southwestern (SRMC) Regional Medical Centers between Jan 2001 and Dec 2008. None of these patients had received any treatment at our hospitals when contacted to participate in this investigation. The inclusion criteria for participation in this study were a histological diagnosis of lung cancer and the ability to read English. Patients with all stages of lung cancer were eligible for the study. Patients were excluded if they were unable to give informed consent or were unable to understand or cooperate with study conditions.

A trained clinical coordinator was responsible for determining eligibility, describing the study, and obtaining informed consent. All patients were assured that refusal to participate would not affect their future care in any way. Patients who chose to participate were presented with the questionnaire at their initial visit and instructed to return their completed questionnaires to the clinical coordinator within 24 hours; thus, patients completed questionnaires prior to receiving therapy at our facility. Data were systematically collected and entered in an excel spreadsheet which was maintained by the Office of Clinical Research. Several quality checks were performed from time to time to ensure completeness and accuracy of data. QoL data used in this study is part of a long-term prospective database on QoL in cancer patients treated at CTCA.

Additional patient data recorded for this study were gender, age at presentation (current age), stage of disease at diagnosis and prior treatment history. The only follow-up information required was the date of death or the date of last contact/last known to be alive. This study was approved by the Institutional Review Boards at MRMC and SRMC.

### QoL Assessment

QoL was assessed using European Organization for the Research and Treatment of Cancer Core Quality of Life Questionnaire (QLQ-C30), which emphasizes a patient's capacity to fulfill the activities of daily living. The QLQ-C30 is a 30-item cancer specific questionnaire that incorporates five functioning scales (physical, role, cognition, emotional, and social), nine symptom scales (fatigue, pain, and nausea/vomiting, dyspnea, insomnia, loss of appetite, constipation, diarrhea, financial problems), and a global health status/QoL scale. The raw scores are linearly transformed to give standard scores in the range of 0-100 for each of the functioning and symptom scales. Higher scores in the global and functioning scales and lower scores in the symptom scales indicate better QoL. A difference of 5-10 points in the scores represents a small change, 10-20 points a moderate change and greater than 20 points a large clinically significant change from the patient's perspective [35]. This instrument has been judged to be reliable and valid as a result of extensive testing in a wide range of clinical cancer populations [36-38].

### Prespecified Baseline Clinical Factors

Baseline clinical factors that were assessed for prognostic significance were gender, current age, stage of disease at diagnosis and prior treatment history. Stage at diagnosis was categorized into two groups consisting of locoregional (stages I-III) and metastatic (stage IV) disease. The prior treatment history variable categorized the patients into those who have received definitive cancer treatment elsewhere before coming to our institution and those who were newly diagnosed at the time of presentation to our institution. These data were obtained from the tumor registries of MRMC and SRMC.

### Data Analysis and Statistical Methods

Patient survival was the primary end point and defined as the time interval between the date of first patient visit to the hospital and the date of death from any cause or the date of last contact/last known to be alive. The overall survival was calculated using the Kaplan-Meier or product-limit method. Clinical and QoL variables were also evaluated using univariate Cox regression analyses to determine which parameters showed individual prognostic value for survival. Multivariate Cox regression analyses were then performed to evaluate the joint prognostic significance of those QoL and clinical factors that were shown to be prognostic in univariate analyses. We used both forward stepwise method as well as the block entry method (all variables entered together at the same time in one block). Forward stepwise method was used because, as is common in QoL data, many of the individual QoL scales are highly correlated. Stepwise regression avoids the problem of multicollinearity because two highly correlated attributes will normally not both be entered in the model. Since the global QoL scale of the QLQ-C30 is highly correlated with other scales, it was not included in prognostic indicator analyses when other variables from QLQ-C30 were used, in order to achieve model stability [28]. Instead, global QoL was analyzed separately after adjusting for clinical and demographic factors. Separate analyses stratified by prior treatment history were conducted to identify QoL factors prognostic of survival in both newly diagnosed and previously treated patients.

Cox regression with time-invariant covariates assumes that the ratio of hazards for any two groups remains constant in proportion over time. We checked this assumption by first examining log-minus-log plots for the categorical predictors and then fitting a Cox regression with a time-varying covariate for each predictor in turn. Each QLQ-C30 scale was treated as a continuous variable for the purpose of Cox regression analyses. The effect of QoL parameters on patient survival was expressed as Hazard Ratios (HRs) with 95% confidence intervals (CIs). Pearson's correlation coefficients were used to investigate the association between different QoL variables. An effect was considered to be statistically significant if the p value was less than or equal to 0.05. All data were analyzed using SPSS version 17.0 (SPSS, Chicago, IL, USA).

## Results

### Patient characteristics

Table 1 describes the baseline characteristics of our patient cohort while Table 2 provides descriptive statistics for the population from the QLQ-C30 scale scores. Among the QLQ-C30 functioning scales, role functioning had the lowest (worst) mean score of 58.9 while the highest (best) mean score of 74.9 was recorded for cognitive functioning. Among the QLQ-C30 symptom scales, diarrhea had the lowest (best) mean score of 9.9 while the highest (worst) mean score of 46.8 was recorded for fatigue.

**Table 1:** Baseline characteristics of 1194 lung cancer patients

Characteristic	Categories	Number	Percent
Age at presentation (years)	■ Mean	58.3	
	■ Median	58.5	
	■ Range	21.6-86.4	
Gender	■ Male	601	50.3
	■ Female	593	49.7
Tumor Stage at Diagnosis	■ Stage 1	100	8.4
	■ Stage 2	63	5.3
	■ Stage 3	348	29.1
	■ Stage 4	656	54.9
	■ Indeterminate	27	2.3
Vital Status	■ Expired	778	65.2
	■ Alive	416	34.8
Treatment History	■ Newly diagnosed	605	50.7
	■ Previously treated	589	49.3

**Table 2:** Baseline QoL scores of 1194 lung cancer patients

QLQ-C30 Scale	Mean	Median	SD	Range
Global	53.6	58.3	26.3	0-100
Physical	68.7	73.3	25.5	0-100
Role	58.9	66.7	34.3	0-100
Emotional	64.1	66.7	25.8	0-100
Cognitive	74.9	83.3	25.9	0-100
Social	61.2	66.7	32.9	0-100
Fatigue	46.8	44.4	28.4	0-100
Nausea/Vomiting	14.5	0	22.2	0-100
Pain	37.1	33.3	32.8	0-100
Dyspnea	37.6	33.3	33.6	0-100
Insomnia	39.9	33.3	33.5	0-100
Appetite Loss	31.3	33.3	34.1	0-100
Constipation	24.2	0	31.3	0-100
Diarrhea	9.9	0	19.7	0-100

Higher scores in the global and functioning scales and lower scores in the symptom scales indicate better QoL.

### Relationship between QoL and other covariates

Table 3 describes the distribution of QoL scores by stage of disease and prior treatment history using 2 sample t-tests. Mean global QoL scores were 53.8 and 53.1 for locoregional and metastatic disease (p = 0.63) and 56.1 and 50.9 for newly diagnosed and previously treated disease respectively (p = 0.001). Fatigue and pain scores were significantly higher (poorer) in patients with metastatic disease as compared to those with locoregional disease. Similarly, physical, role and social functioning scores and fatigue, nausea/vomiting, pain, dyspnea and appetite loss symptom scores were significantly poorer in previously treated patients as compared to newly diagnosed patients. We also evaluated the relationship between QoL and age at presentation using Pearson correlation (r). The function scales to be significantly correlated with age were physical (r = -0.06; p = 0.04), emotional (r = 0.07; p = 0.01) and social (r = 0.07; p = 0.01). Physical scale was negatively correlated (such that higher age was associated with a poorer physical function) while emotional and social scales were positively correlated (such that higher age was associated with better emotional and social functions). Among the symptom scales, nausea/vomiting (r = -0.09; p = 0.001), pain (r = -0.16; p = 0.001) and insomnia (r = -0.12; p = 0.001) were negatively correlated with age (such that higher age was associated with better nausea/vomiting, pain and insomnia) while dyspnea (r = 0.08; p = 0.007) was positively correlated (such that higher age was associated with poorer dyspnea). All these correlations were very weak (with r less than 0.20 in either direction).

**Table 3:** Distribution of QoL scores by stage of disease and prior treatment history

QLQ-C30 Scale	Stage of disease			Treatment History		
	Locoregional (N = 511)	Metastatic (N = 656)	P	Newly diagnosed (N = 605)	Previously treated (N = 589)	P
Global	53.8	53.1	0.63	56.1	50.8	**0.001**
Physical	69.4	68.0	0.35	73.1	64.1	**< 0.001**
Role	60.4	57.2	0.12	62.4	55.2	**< 0.001**
Emotional	64.5	63.5	0.55	63.3	64.8	0.33
Cognitive	75.1	74.5	0.68	75.9	73.7	0.16
Social	62.0	60.2	0.35	65.3	56.8	**< 0.001**
Fatigue	45.0	48.6	**0.03**	43.1	50.6	**< 0.001**
Nausea/Vomiting	13.9	15.1	0.36	12.2	16.8	**< 0.001**
Pain	34.7	39.0	**0.02**	34.0	40.3	**0.001**
Dyspnea	39.5	36.0	0.08	34.7	40.6	**0.002**
Insomnia	39.0	41.0	0.31	40.5	39.3	0.54
Appetite Loss	30.2	32.3	0.29	28.4	34.1	**0.004**
Constipation	22.7	25.4	0.14	23.2	25.2	0.27
Diarrhea	9.9	9.8	0.92	8.8	10.9	0.07

Higher scores in the global and functioning scales and lower scores in the symptom scales indicate better QoL.

### Univariate analysis: prognostic factors for overall survival

Median overall survival for the entire patient cohort was 8.8 months (95% CI: 8.0-9.5 months). The median survival for newly diagnosed and previously treated patients was 12.6 and 6.8 months respectively, p < 0.001. The median survival for patients with locoregional and metastatic disease was 12.0 and 7.5 months respectively, p < 0.001. Table 4 describes the results of univariate Cox regression analyses for each QLQ-C30 scale as well as age, stage of disease and prior treatment history. The HRs along with their 95% CIs for every one-unit increase in all QLQ-C30 scales are given. On univariate analysis, QoL scales predictive of survival were global (p < 0.001), physical (p < 0.001), role (p < 0.001), emotional (p = 0.04), cognitive (p = 0.003), social (p < 0.001), fatigue (p < 0.001), nausea/vomiting (p = 0.001), pain (p < 0.001), dyspnea (p < 0.001), loss of appetite (p < 0.001), constipation (p = 0.001) and diarrhea (P = 0.02). Gender, stage of disease at diagnosis and prior treatment history were also found to be significant predictors of survival upon univariate analysis (p < 0.001 for all).

**Table 4:** Univariate Cox regression analysis for overall survival

QoL Domain	HR	95% CI	P-value
Global	0.991	0.989 to 0.994	**< 0.001**
Physical	0.988	0.985 to 0.990	**< 0.001**
Role	0.993	0.991 to 0.995	**< 0.001**
Emotional	0.997	0.994 to 1.00	**0.04**
Cognitive	0.996	0.993 to 0.999	**0.003**
Social	0.994	0.992 to 0.996	**< 0.001**
Fatigue	1.009	1.006 to 1.011	**< 0.001**
Nausea/vomiting	1.005	1.002 to 1.008	**0.001**
Pain	1.006	1.004 to 1.009	**< 0.001**
Dyspnea	1.005	1.003 to 1.008	**< 0.001**
Insomnia	1.000	0.998 to 1.003	0.66
Appetite Loss	1.006	1.004 to 1.008	**< 0.001**
Constipation	1.004	1.002 to 1.006	**0.001**
Diarrhea	1.004	1.001 to 1.008	**0.02**
Age at presentation	0.998	0.991 to 1.006	0.65
Gender (male as reference)	0.77	0.67 to 0.89	**< 0.001**
Stage at diagnosis (locoregional disease as reference)	1.57	1.4 to 1.8	**< 0.001**
Prior treatment history (previously treated as reference)	0.55	0.48 to 0.64	**< 0.001**

### Multivariate analysis: prognostic factors for overall survival

Table 5 describes the results of multivariate Cox regression analyses using the block entry method for those QLQ-C30 function and symptom scales that were significant upon univariate analysis after controlling for the effects of gender, tumor stage and prior treatment history. Upon multivariate analyses, only physical function (p < 0.001) was predictive of survival independent of other QoL scales, gender, stage, and treatment history. Every 10-point increase in physical function was associated with a 10% increase in survival (95% CI = 6% to 14%, p < 0.001). Gender, stage of disease and prior treatment history were also found to be significant predictors in the final multivariate model (p < 0.01 for all). The findings described above were confirmed using the forward stepwise method. It was interesting to see that most of the function and symptom scales that were predictive of survival upon univariate analysis lost their statistical significance upon multivariate analysis. This is not an uncommon observation in QoL analyses, because many of the individual QoL scales are highly correlated with each other, and therefore lose their statistical significance when analyzed together.

**Table 5:** Multivariate Cox regression analysis for QoL function and symptom scales (Block Entry Method)

QoL Domain	HR	95% CI	P-value
Physical	0.990	0.986 to 0.994	**< 0.001**
Role	0.999	0.995 to 1.002	0.50
Emotional	1.001	0.997 to 1.005	0.58
Cognitive	1.000	0.996 to 1.003	0.80
Social	1.000	0.996 to 1.003	0.85
Fatigue	0.996	0.992 to 1.001	0.16
Nausea/vomiting	0.997	0.993 to 1.001	0.20
Pain	1.002	0.999 to 1.005	0.16
Dyspnea	1.001	0.999 to 1.004	0.34
Appetite Loss	1.003	1.000 to 1.005	0.07
Constipation	1.000	0.998 to 1.003	0.73
Diarrhea	1.003	0.999 to 1.006	0.16
Gender (male as reference)	0.78	0.67 to 0.90	**0.001**
Stage at diagnosis (locoregional disease as reference)	1.67	1.4 to 1.9	**< 0.001**
Prior treatment history (previously treated as reference)	0.55	0.47 to 0.64	**< 0.001**

Table 6 describes the results of multivariate Cox regression analyses using the block entry method for global QoL after controlling for the effects of gender, tumor stage and prior treatment history. Every 10-point increase in global QoL was associated with a 9% increase in survival (95% CI = 6% to 11%, p < 0.001). Gender, stage of disease and prior treatment history were also found to be significant predictors in the final multivariate model (p < 0.01 for all).

**Table 6:** Multivariate Cox regression analysis for global QoL (Block Entry Method)

QoL Domain	HR	95% CI	P-value
Global	0.991	0.989 to 0.994	**< 0.001**
Gender (male as reference)	0.78	0.68 to 0.91	**0.001**
Stage at diagnosis (locoregional disease as reference)	1.72	1.5 to 1.9	**< 0.001**
Prior treatment history (previously treated as reference)	0.52	0.45 to 0.61	**< 0.001**

## Discussion

Even though the QoL of patients is considered a critical endpoint in oncology that can also provide useful prognostic information to patients and clinicians, QoL continues to be evaluated infrequently in most clinic oncology practices apart from research studies. The barrier is not a lack of valid tools but rather the challenges inherent in incorporating QoL measurements into busy clinical practices [5]. This will continue as long as QoL assessment is regarded as peripheral to the goal of standard clinical cancer therapy. It is our contention, however, that QoL is as meaningful to patients as length of life, a situation which is coming to be appreciated by patients, advocates, insurance providers, and employers. Thus, there is a strong rationale to incorporate regular QoL assessment and management into all oncologic practice settings.

In the current study, we chose QLQ-C30 as a valid and reliable tool to assess patient QoL which is appropriate for assessing large, heterogeneous patient populations. It is relevant that this instrument concentrates on a patients' ability to fulfill the activities of daily living. While this emphasis has been regarded in research studies as an appropriate way to assess the effectiveness of new drugs or novel combinations of agents, cumulative experience from several decades of use of the QLQ-C30 also demonstrate the relevance of this information for clinical practitioners. Thus, results from the QLQ-C30 provide important information to guide treatment decisions based on balancing the need to control tumor progression with the need to know how treatment affects the patient's capacity to fulfill the activities of their daily life at work and in the home.

In this study, we found that global QoL as well as physical function were significant predictors of patient survival in the entire study population. The finding of physical function predicting survival in lung cancer has been reported by previous studies [11,20,39-41]. For example, Ganz et al. reported a statistically significant relationship between initial patient-rated QoL scores and subsequent survival [40]. In another study by Herndon et al., increased pain, appetite loss, fatigue, lung carcinoma symptoms, poorer overall QoL, and poorer physical functioning predicted significantly poorer survival. However, only the pain score was retained in the final multivariate model [20]. Finally, a study conducted by Fielding and Wong found that better physical functioning and better appetite were predictive of longer survival after fully adjusting for sociodemographic and clinical factors in Chinese lung cancer patients [39].

The finding of global QoL predicting survival in lung cancer has also been reported by previous studies [15,20,22,25,41-43]. For example, Langendijk et al. examined the significance of pretreatment QoL and symptom scores in lung cancer patients treated with high dose radiotherapy and found the EORTC global QoL score as the strongest prognostic factor [22]. Montazeri et al. showed that pre-diagnosis EORTC global QoL was a significant predictor of survival in lung cancer [25]. Dharma-Wardene et al. found that baseline FACT-G total score is a statistically significant predictor of survival in patients with advanced lung cancer [15]. Brown et al. found EORTC global QoL, role functioning, fatigue, appetite loss, and constipation as prognostic indicators of survival in patients with NSCLC at 12 weeks [42]. Maione et al. found that only pretreatment EORTC global QoL and instrumental activities of daily living scores were significant predictors for survival in elderly patients with advanced non-small-cell lung cancer treated with chemotherapy [43].

In the previously published studies mentioned above, the most commonly adjusted variables were age, gender, socioeconomic status, performance status, various cell counts, metastasis, treatment types, cancer stage, histology subtypes, sites involved, and weight loss. However, it is worth mentioning that some of these studies failed to provide adequate controls for confounding, for example, there is no information on the extent of the disease or whether the tumor size shrank in response to therapy [15,22,32]. In most of these studies, it is not possible to determine whether QoL independently predicted survival or simply reflected the progress of the underlying disease. For example, it has been suggested that physical functioning might be a surrogate marker for an unrecognized biological prognostic indicator, so a causal association between physical functioning and survival time should not be inferred [28].

It is to be noted that in our study, several QoL function and symptom scales that were predictive of survival on univariate analysis, lost their statistical significance after the basic demographic and clinical factors were adjusted for. Only physical and global function retained their statistical significance in multivariate modeling. Collectively, these results demonstrate that after the potential confounding factors are adjusted for in the multivariate analysis, the prognostic effects of QoL either disappear or are mitigated significantly. Thus, before making any claims regarding the independent prognostic effects of QoL in cancer, adequate statistical adjustment of covariates needs to be achieved. Finally, our finding on global function should be interpreted with caution because the global function score likely reflects the contribution of the physical function score.

We also found that physical function, nausea/vomiting, insomnia, and diarrhea in newly diagnosed patients predicted survival in multivariate analysis. Similarly, in previously treated patients, physical function was predictive of survival in multivariate analysis. For both of these clinical settings, it is easy to appreciate the negative impact these attributes would exert on the ability of a patient to function. While the current study does not elucidate causative relationships between these functions and patient survival, the importance of measuring these functions in patients and attempting to address their effects therapeutically should be obvious. The finding of poorer physical, role and social functioning scores and fatigue, nausea/vomiting, pain, dyspnea and appetite loss symptom scores in previously treated patients as compared to newly diagnosed patients is not surprising given that previously treated patients have more advanced disease and are well into their course of illness as compared to newly diagnosed patients.

The results of this study have important implications for both clinical and research practices in an integrative oncologic care setting. They suggest that health care professionals should evaluate baseline QoL in all patients and take this into consideration when planning treatment. QoL should also be assessed regularly during treatment and appropriate intervention taken to improve QoL when indicated. QoL was initially designed to be used as a clinical indicator. Self-reported QoL assessment using validated questionnaires plays a very important role in acquiring and monitoring information on patient's functional and symptom status throughout the course of treatment. In the absence of concrete information on the prognostic role of QoL in cancer survival, its utility as a clinical indicator should not be jeopardized. In fact, information about QoL in lung cancer patients undergoing treatment can provide healthcare providers with a perspective on post-treatment recovery, including the positive aspects of long-term care, as well as anticipated problems [3].

Although this study presents interesting findings and additional questions for further inquiry, several limitations require acknowledgment. Our study, because of its retrospective nature, relies on data not primarily meant for research. As a result, we could not control for some additional factors in our analyses that could influence survival such as medical comorbidities, performance status, socioeconomic factors, support system, exercise, insurance coverage, employment and educational level. It has been suggested that QoL data might be markers of the socioeconomic status of cancer patients. For instance, cancer patients with higher social class would have a better QoL, and consequently those who report a better QoL at baseline assessment may live longer [28]. As suggested by Fielding and Wong, full adjustment is needed for factors affecting outcome in order to demonstrate any independent predictive power of QoL [39]. The patient cohort was limited only to those patients who were English speakers and therefore is not representative of the complete spectrum of lung cancer patients. A majority of our patients had advanced stage disease at presentation. As a result, generalizability of the study findings to NSCLC patients with early stage disease might be questionable. However, we have no reasons to believe that patients with early stage disease will have different findings. Moreover, this study is not able to reveal causative relationships between any QoL element and patient survival. Rather, QoL was found to act as a surrogate marker for otherwise undetected prognostic factors [11]. We did not control for the multiple comparisons made in this study, but this is acceptable for hypothesis-generating studies [26]. Finally, we did not evaluate the prognostic significance of changes in QoL scores during the entire duration of treatment.

Nevertheless, this study also has several strengths, including a large sample size; complete data for all QLQ-C30 subscales for the entire study sample; high compliance with completion of the questionnaire; the use of a valid and reliable QoL instrument; comprehensive documented clinical parameters in nearly all patients; and availability of mature and reliable survival data.

## Conclusions

This study suggests that baseline global QoL and physical function scale provide useful prognostic information in non-small cell lung cancer patients.

## Competing interests

The authors declare that they have no competing interests.

## Authors' contributions

DPB and DG participated in concept, design, data collection, data analysis, data interpretation and writing. EDG participated in concept, design and data interpretation. All authors read and approved the final manuscript.

## Pre-publication history

The pre-publication history for this paper can be accessed here:

http://www.biomedcentral.com/1471-2407/11/353/prepub
